# Microbiota-Mediated Bile Acid Metabolism as a Mechanistic Framework for Precision Nutrition in Gastrointestinal and Metabolic Diseases

**DOI:** 10.3390/cells15010023

**Published:** 2025-12-22

**Authors:** Suna Kang, Do-Youn Jeong, Jeowon Seo, James W. Daily, Sunmin Park

**Affiliations:** 1 Department of Food and Nutrition, Obesity/Diabetes Research Center, College of Biohealth Science, Hoseo University, Asan-si 31499, Republic of Korea; 2 Microbial Institute for Fermentation Industry, Sunchang-gun 56048, Republic of Korea; jdy2534@korea.kr (D.-Y.J.);; 3 R&D, Daily Manufacturing, Rockwell, NC 28138, USA; jdaily3@yahoo.com

**Keywords:** gut microbiota, enterotypes, bile acids, host genetic polymorphism

## Abstract

Gut microbiota play a central role in shaping bile acid (BA) metabolism through community-specific capacities for deconjugation, dehydroxylation, and other transformation reactions. Distinct microbiome compositional patterns—often referred to as enterotype-like clusters—correspond to reproducible functional profiles that generate unique BA metabolic signatures with relevance for metabolic and gastrointestinal health. This narrative review synthesizes current evidence describing the interplay between microbial composition, BA metabolism, and metabolic dysfunction. A structured literature search was conducted in PubMed, Web of Science, EMBASE, and Scopus using predefined keywords related to bile acids, microbiome composition, metabolic disorders, and enterotypes. Studies were screened for human clinical relevance and mechanistic insights into BA–microbiome interactions. Across the evidence base, *Bacteroides-*, *Prevotella*-, and *Ruminococcus*-associated community types consistently demonstrate different BA transformation capacities that influence secondary BA production and downstream host signaling through *FXR* and *TGR5*. These differences are linked to variation in metabolic dysfunction-associated steatotic liver disease, obesity, type 2 diabetes, inflammatory bowel disease, and colorectal cancer. Host genetic variations in BA synthesis, transport, and signaling further modify these microbiome–BA interactions, contributing to the heterogeneity of dietary intervention responses. Overall, the literature supports a model in which microbiome-derived BA profiles act as metabolic phenotypes that shape host lipid and glucose homeostasis, inflammation, and gut–liver axis integrity. Emerging clinical applications include microbiome-stratified dietary strategies, targeted probiotics with defined BA-modifying functions, and therapeutic approaches that align BA-modulating interventions with an individual’s microbial metabolic capacity. Establishing integrated biomarker platforms combining microbiome clustering with BA profiling will be essential for advancing precision nutrition and personalized management of metabolic and gastrointestinal diseases.

## 1. Introduction

The human gut microbiome harbors trillions of microorganisms that profoundly influence host physiology, metabolism, and disease susceptibility [1]. Among the frameworks proposed to interpret this complexity, compositional clustering of gut microbiota—an analytical approach that identifies distinct, reproducible patterns in microbial community structure—has emerged as a powerful model for understanding inter-individual variation in microbiome function [1]. These compositional clusters, commonly referred to as enterotypes, were first formally described by Arumugam et al. in 2011 [2]. Enterotypes represent relatively stable microbial configurations dominated by *Bacteroides*, *Prevotella*, or *Ruminococcus* genera. Although enterotype distributions vary across populations, these data-driven clusters consistently align with differences in microbial metabolic capacity, especially in bile acid (BA) transformation.

Enterotypes exhibit distinguishable enzymatic signatures related to bile salt hydrolase activity, deconjugation capacity, and 7α-dehydroxylation potential encoded by the BA-inducible (*bai*) operon [3]. *Bacteroides*-dominated communities (ET-B) typically display high deconjugation capacity but limited secondary BA production, whereas *Prevotella*-enriched communities (ET-P) emphasize carbohydrate fermentation. *Ruminococcus*-associated enterotypes (ET-R) harbor extensive secondary BA-producing machinery, generating metabolites with potent effects on *FXR* and *TGR5* signaling. Because enterotype characteristics remain relatively stable over months to years [4,5], these differences may contribute to the 30–60% variability in metabolic responses to dietary interventions, which are usually designed based on population-averaged data [6].

Growing evidence links enterotype-specific BA profiles to metabolic dysfunction-associated steatotic liver disease (MASLD), obesity, type 2 diabetes, inflammatory bowel disease, and neuroinflammatory conditions [7,8,9,10]. As BAs act not only as digestive molecules but also as key metabolic and immune regulators, differences in microbiome-mediated transformation influence disease susceptibility and therapeutic responsiveness [11]. However, most existing reviews address enterotypes and BA metabolism separately or focus on specific diseases, leaving an important gap: a comprehensive synthesis of how microbial community structure, enzymatic capacity, and BA signaling interact to shape dietary and metabolic outcomes.

While enterotypes provide a practical framework for linking microbiome composition with metabolic function, they should not be interpreted as rigid or mutually exclusive categories [12]. Early studies emphasized *Bacteroides*- (ET-B) and *Prevotella*-dominated (ET-P) enterotypes; however, subsequent analyses identified a third community type often characterized by *Ruminococcus* and related taxa within the Lachnospiraceae family, sometimes described as a *Lachnospira*-associated enterotype (ET-L) [7,13,14]. Beyond these canonical groupings, accumulating evidence indicates that gut microbial communities frequently exhibit intermediate or mixed configurations rather than discrete *Bacteroides*-, *Prevotella*-, or *Ruminococcus*-dominant states [15]. Accordingly, enterotypes are best viewed as probabilistic metabolic phenotypes—a functional framework that bridges host genetics, microbial ecology, and BA-mediated metabolic regulation. This continuum-based perspective helps explain substantial inter-individual variability in dietary and metabolic responses and positions enterotypes as an informative. However, it is an incomplete intermediate step toward fully personalized, microbiome-informed nutrition [16].

This review fills this evidence gap by integrating mechanistic, clinical, and translational perspectives to establish a unified framework for enterotype–BA interactions and their relevance to precision nutrition. Specifically, we: (1) summarize determinants of enterotype stability, (2) describe functional differences in BA transformation across community types, (3) evaluate their relevance to metabolic and gastrointestinal diseases, (4) explain how these differences drive inter-individual variability in dietary response, and (5) propose clinically applicable strategies for microbiome-stratified nutrition.

To ensure comprehensive coverage, a literature search was conducted in PubMed, Web of Science, and Scopus using combined keywords related to gut microbiota, enterotypes, BA metabolism, metabolic diseases, and gut–liver axis physiology. Both human studies and mechanistic animal studies were included when they provided insight into microbial BA transformation and host metabolic regulation. Additional relevant articles were identified through a manual review of the reference lists. This review is narrative in nature, integrating mechanistic and clinical evidence to address the conceptual gap described above.

## 2. Gut Microbiota Enterotypes: Compositional Patterns and Functional Implications

### 2.1. Definition, Historical Context, and Classification Principles

The concept of gut microbiota enterotypes emerged from the recognition that human gut microbial communities cluster into distinct, functionally coherent configurations despite substantial inter-individual variability. First described by Arumugam et al. [2] through metagenomic analysis, enterotypes represent stable microbial community structures characterized by dominant bacterial genera and their associated metabolic networks [1]. Three primary clusters were initially identified: the ET-B, ET-P, and ET-R (ET-L) enterotypes [2,12,13].

Beyond taxonomy, enterotypes represent functionally distinct metabolic ecosystems with differential capacities for carbohydrate degradation, amino acid fermentation, and BA transformation [17]. These functional profiles underpin diverse host–microbe interactions and explain why identical dietary interventions lead to divergent metabolic outcomes across individuals. Increasing evidence suggests that enterotypes exist not as discrete categories but as stable attractor states within a continuous microbial landscape, maintained through self-reinforcing feedback loops between the diet, host genetics, and microbial metabolism [15].

### 2.2. Characteristics of the Major Human Enterotypes

#### 2.2.1. Characteristics of ET-B

ET-B is characterized by a high abundance of *Bacteroides* species, Gram-negative anaerobes possessing extensive carbohydrate-active enzyme (CAZyme) repertoires that enable the degradation of diverse polysaccharides. Individuals with ET-B typically harbor over 260 CAZyme families, conferring the metabolic flexibility to process both host-derived mucins and dietary fibers [18]. This versatility reflects an evolutionary adaptation to protein- and fat-rich Western diets.

BA Metabolism Profile: ET-B exhibits robust bile salt hydrolase (BSH) activity, enabling efficient deconjugation of primary BAs. *Bacteroides* species (spp.) possess multiple BSH enzymes with broad substrate specificity, thereby influencing the primary-to-secondary BA ratio and shaping gut microbial ecology [19]. This extensive BA transformation results in elevated fecal BA excretion, as secondary BAs exhibit significantly lower reabsorption efficiency (via ASBT) compared to primary BAs. Consequently, ET-B individuals typically demonstrate high BA turnover rates, with compensatory hepatic synthesis maintaining pool homeostasis [20]. These BA transformations reinforce *Bacteroides* dominance while modulating the colonization patterns of other taxa. Clinical Associations: The evidence linking ET-B to metabolic disorders is inconsistent. Bacteroides abundance has been reported to be both increased in obesity and decreased in metabolic dysfunction across different studies [21]. Notably, reduced *Bacteroides* abundance is frequently observed in MASLD, and several *Bacteroides* spp. have demonstrated therapeutic potential in ameliorating hepatic steatosis and insulin resistance [19]. The *Bacteroides* 2 (ET-B2) subtype, however, is particularly linked to metabolic syndrome and obesity [16,22]. ET-B also modulates toll-like receptor 4 (TLR4) signaling through the lipopolysaccharide (LPS)-mediated pathways [23]. The precise relationship between *Bacteroides* abundance, gut barrier integrity, and TLR4 activation remains under investigation, with emerging evidence suggesting situation-dependent protective or pathogenic roles, depending on host factors and microbial strain composition.

#### 2.2.2. Characteristics of ET-P

ET-P is dominated by *Prevotella* species that specialize in fermenting plant-derived polysaccharides, reflecting adaptation to the fiber-rich diets typical of non-Westernized populations [23,24]. BA Metabolism Profile: ET-P demonstrates limited direct BA transformation capacity compared to ET-B. While *P. copri* exhibits modest BSH activity, it lacks the extensive 7α-dehydroxylase capability characteristic of *Bacteroides* and Clostridiales species [25]. Consequently, ET-P maintains a higher proportion of primary BAs (CA, CDCA) in their conjugated forms, which are efficiently reabsorbed via ASBT in the terminal ileum, resulting in lower fecal BA excretion and reduced BA pool turnover [26]. However, ET-P’s metabolic influence on BA homeostasis operates through indirect mechanisms. *P. copri* may create an intestinal environment that modulates the activity of secondary BA-producing Clostridiales when present, potentially affecting the ratio of CDCA derivatives. More significantly, ET-P’s characteristic high SCFA production—particularly acetate and propionate—creates acidic intestinal conditions that influence BA solubility, micelle formation, and may attenuate FXR signaling [27,28]. Additionally, SCFAs can modulate intestinal transit time and affect the kinetics of BA exposure to the intestinal epithelium. Clinical Associations: ET-P displays strain-dependent metabolic effects. Certain *P. copri* strains associated with high-fiber diets correlate with improved glucose homeostasis and anti-inflammatory effects [27,29]. In contrast, other *P. copri* strains have been implicated in Th17-mediated inflammation, rheumatoid arthritis, insulin resistance, and MASLD [30,31,32,33]. These contrasting effects underscore the strain-level functional heterogeneity within ET-P populations.

#### 2.2.3. Characteristics of ET-R

ET-R represents a metabolic intermediate phenotype, combining features of both ET-B and ET-P. It is associated with efficient fiber fermentation, balanced SCFA production, and metabolic stability in individuals consuming mixed diets [17,34]. BA Metabolism Profile: ET-R exhibits moderate BSH activity with especially effective cholate deconjugation activity. *Ruminococcus* spp. contribute to unique BA modification pathways, including the production of iso-BAs (such as iso-DCA and iso-LCA) through enzymatic modifications distinct from classical 7α-dehydroxylation [12,35]. These modified BAs may exhibit altered receptor binding profiles and reabsorption kinetics, potentially contributing to stable BA pool homeostasis with intermediate turnover rates. Additionally, certain *Ruminococcus* species can sequester BAs via bacterial surface binding, which may temporarily reduce their bioavailability for enterohepatic recycling, though the quantitative impact on overall BA homeostasis remains under investigation [35]. Clinical Associations: Elevated butyrate production by *Ruminococcus* spp. supports colonic health, enhances intestinal barrier function, and may mitigate BA-induced inflammation, particularly from hydrophobic secondary BAs [36]. The interplay between iso-BA production, BA sequestration, and SCFA-mediated intestinal conditioning creates a unique metabolic signature that distinguishes ET-R from both ET-B and ET-P.

### 2.3. Enterotype Stability and Classification Challenges

A major challenge in enterotype research lies in determining whether they represent discrete biological entities or points along a continuum of microbial variation. Large-scale analyses support statistically discernible clustering. However, the boundaries between enterotypes are gradual rather than sharp [23]. Temporal Stability: Longitudinal studies have reported that approximately 80–85% of individuals maintain their dominant enterotype over time despite dietary fluctuations, largely sustained by BA–microbe feedback mechanisms [23,37]. Nonetheless, major dietary shifts, antibiotic exposure, or disease can trigger transitions, often mediated through BA-driven ecological restructuring. Methodological Considerations: Enterotype identification is influenced by clustering algorithms, taxonomic resolution, and distance metrics. Genus-level analyses may overlook species- or strain-specific functional differences critical for BA metabolism. Integrating metabolic pathway and gene content analyses provides a more robust framework for understanding enterotype-related metabolic functions [12,38].

### 2.4. Host Genetic and Environmental Determinants of Enterotypes

Host genetics account for roughly 10–20% of gut microbiome variation, with significant implications for BA metabolism [39]. Key genetic determinants include the following: (1) BA Synthesis Genes: Polymorphisms in cytochrome P450 family 7 subfamily A member 1 (*CYP7A1*) and cytochrome P450 family 8 subfamily B member (*CYP8B1*) influence primary BA composition and thus shape microbial community structure. Reduced CYP8B1 activity leads to CDCA-enriched bile pools, favoring the *Clostridium* and *Bacteroides* species associated with enhanced insulin sensitivity [39,40,41]. (2) BA Transport and Signaling: Variants in solute carrier organic anion transporter family member 1B1 (*SLCO1B1*), ATP-binding cassette subfamily B member 1 (*ABCB1*), and nuclear receptors *FXR* and *TGR5* modulate BA transport and feedback signaling, thereby creating host-specific selective pressures for establishing the enterotype [42,43,44,45]. (3) Dietary determinants: High dietary fat intake stimulates BA production and secretion, creating selective conditions favoring the *Bacteroides* spp. with robust BSH activity. The resulting increase in secondary BA production further reinforces ET-B dominance through antimicrobial effects on BA-sensitive taxa [46]. (4) Fiber and Carbohydrate Composition: The consumption of complex carbohydrates may promote *Prevotella* proliferation through metabolic substrates, while also reducing requirements for BA production. The resulting low BA concentration is associated with microbial communities that are involved in polysaccharide fermentation rather than BA modification. [47,48]. Micronutrient Cofactors: Dietary availability of the cofactors required for BA metabolism (particularly B-vitamins and minerals) influences the efficiency of the microbial BA transformation pathways, contributing to enterotype stability and metabolic output patterns [49]. The complex interplay between host genetics, diet, and BA metabolism establishes individualized ecological niches that determine the enterotype composition and metabolic function, forming the foundation for personalized nutrition and microbiome-based therapeutics [50].

## 3. BA Composition Across Enterotypes: From Hepatic Synthesis to Enterohepatic Circulation

This section synthesizes current evidence on BA synthesis, transport, and signaling pathways, alongside microbial transformations that shape the BA pool. Bacterial BA metabolism—including deconjugation, dehydroxylation, and epimerization—within the broader context of BA–microbiota networks as well as host regulatory mechanisms. Factors influencing microbial BA metabolism, such as gut transit time, dietary composition, host genetics, and enterotype-specific microbial functions, are integrated throughout the review. By organizing the content across enterotypes, we provide a framework that connects BA production and enterohepatic circulation (EHC) with microbial functional capacities and their implications for metabolic health.

### 3.1. Hepatic BA Synthesis: Classical and Alternative Pathways

**Figure 1:**
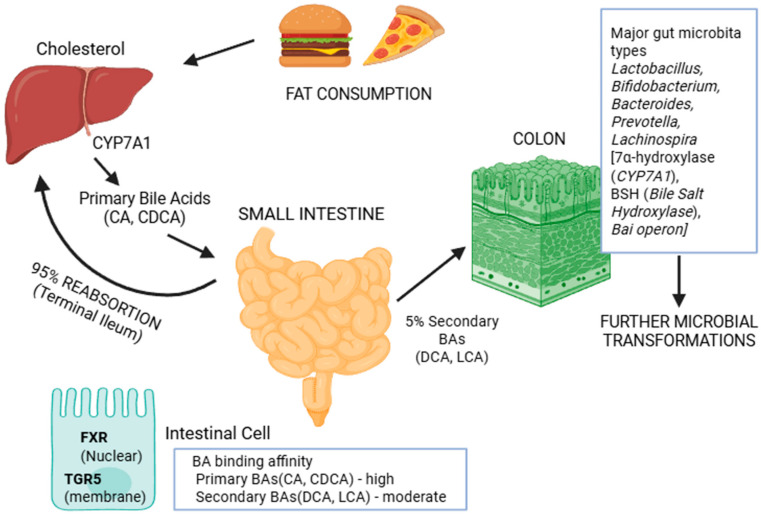
Integrated bile acid metabolism and gut microbial axis.

Hepatic BA synthesis is the primary route of cholesterol catabolism and a central determinant of BA pool composition, thereby shaping both microbial ecology and host metabolic signaling [5]. Two biosynthetic pathways—the classical (neutral) and alternative (acidic)—operate under differential regulatory control in response to metabolic and inflammatory cues (Figure 1) [5].

The classical pathway, responsible for approximately 75–90% of total BA synthesis, is initiated by cholesterol 7α-hydroxylase (encoded by *CYP7A1*), the rate-limiting enzyme tightly regulated by *FXR*, liver X receptor (*LXR*), and hepatocyte nuclear factor 4α (*HNF4α*) (Figure 1) [51]. Microbial transformation of BAs plays an essential feedback role, as secondary BAs exhibit greater *FXR*-binding affinity than primary BAs, thereby modulating the intensity of hepatic synthesis. The pathway diverges at sterol 12α-hydroxylase encoded by *CYP8B1*, which determines the ratio of cholic acid (CA) and CDCA. Variations in genetic polymorphisms of *CYP8B1* or its activity alter this ratio, which may predispose individuals to distinct enterotype configurations by creating BA environments with differing microbial selectivity [6]. The alternative (*CYP27A1*-initiated) pathway contributes the remaining 10–25% of BA synthesis and becomes particularly active when classical pathway activity is suppressed. This pathway favors CDCA production, exhibits resistance to *FXR*-mediated inhibition, and often compensates during inflammatory or metabolic stress [52,53]. Such shifts in BA composition influence colonic microbial selection, promoting the growth of taxa with high BA tolerance [54].

Following synthesis, primary BAs are conjugated with glycine (~75%) or taurine (~25%) via bile acid CoA synthetase (*BACS*) and BA CoA: amino acid N-acyltransferase (*BAAT*). Conjugation enhances solubility, reduces cytotoxicity, and prevents premature absorption, creating substrates for microbial BSH. The conjugation ratio with glycine and taurine exhibits considerable inter-individual variation, which critically governs substrate availability for microbial deconjugation and downstream BA biotransformations [55,56].

### 3.2. EHC and Enterotype-Specific Patterns

The gut–liver–BA axis forms an intricate communication system linking hepatic metabolism, intestinal microbial activity, and host metabolic regulation. This bidirectional feedback network maintains BA homeostasis and integrates dietary and microbial cues into systemic metabolic responses [57]. Primary BAs synthesized in the liver not only facilitate lipid digestion but also act as ecological regulators, shaping gut microbial composition through their antimicrobial and signaling properties [58]. Conversely, microbially transformed secondary BAs function as endocrine messengers that provide feedback to the liver to regulate BA synthesis and lipid metabolism [51]. Thus, the enterohepatic system operates as a self-reinforcing metabolic circuit, where disturbances in BA signaling—whether through genetic variation, diet, or disease—can propagate across both hepatic and microbial domains.

The EHC recycles a 2–4 g BA pool approximately 4–12 times per day, representing one of the most efficient metabolic recycling processes in humans [59]. Reabsorption occurs predominantly in the terminal ileum via the apical sodium-dependent BA transporter (ASBT encoded by *SLC10A2*), which selectively transports conjugated BAs [60]. Polymorphisms in *SLC10A2* modify reabsorption efficiency, influencing the proportion of BAs entering the colon for microbial transformation [61]. Within enterocytes, ileal BA-binding protein or fatty acid-binding protein 6 (IBABP/FABP6) facilitates intracellular trafficking, while the organic solute transporter (OST-α/β/SLC51A/B) mediates basolateral efflux to portal circulation [60,62].

Microbial BSH activity deconjugates BAs, reducing ASBT affinity and enhancing their escape into the colon for further microbial modification [52]. The extent of deconjugation and subsequent transformation differs markedly across enterotypes: ET-B exhibits high BSH and 7α-dehydroxylase activity (*bai* operon), producing abundant secondary BAs such as DCA and LCA; ET-P demonstrates limited transformation capacity, preserving predominantly primary BAs; and ET-R displays moderate BSH activity with unique iso-BA production pathways [19]. Critically, secondary BAs display significantly lower reabsorption efficiency (50–70%) than primary BAs (>95%), directly modulating BA pool turnover and stimulating compensatory hepatic synthesis [60,63].

Enterotype-specific microbial communities establish characteristic EHC efficiency profiles with distinct BA pool dynamics. Under physiological conditions, approximately 95% of the total BA pool is reabsorbed via ASBT, while 3–5% is excreted in feces—predominantly secondary BAs produced by colonic bacteria [5]. However, this population-average figure masks substantial enterotype-dependent variation driven by differential microbial transformation capacity.

ET-B, characterized by elevated BSH activity and extensive 7α-dehydroxylase expression, generates abundant poorly reabsorbed secondary BAs (DCA, LCA). This results in fecal BA excretion potentially exceeding 5–7% of the BA pool, triggering compensatory hepatic synthesis to maintain pool size and establishing characteristically high BA turnover rates (potentially 8–15 cycles/day) [64,65]. The high turnover in ET-B is primarily driven by the substantially lower reabsorption efficiency of secondary BAs (50–70%) compared to primary BAs (>95%) [63]. Despite high fecal loss, the absolute BA pool size is maintained through this compensatory synthesis, though the pool composition is enriched in secondary BAs.

Conversely, ET-P exhibits limited BA transformation capacity, preserving a higher proportion of efficiently reabsorbed primary BAs in their conjugated forms. This results in lower fecal BA excretion (potentially 2–4% of pool), reduced compensatory synthesis demands, and lower overall BA turnover rates (potentially 4–8 cycles/day). However, ET-P’s characteristic high SCFA production creates acidic intestinal conditions that may influence BA solubility, protonation states, and micellar behavior, potentially affecting both absorption kinetics and FXR/TGR5 receptor activation [28,38]. The interplay between preserved primary BA composition and SCFA-mediated intestinal conditioning creates a distinct metabolic signature.

ET-R manifests intermediate BA pool dynamics. *Ruminococcus*-mediated production of iso-bile acids introduces structurally modified BAs with potentially altered reabsorption efficiencies and receptor binding profiles compared to classical primary and secondary BAs. The reported BA sequestration capacity of certain *Ruminococcus* species may transiently reduce bioavailable BA concentrations during intestinal transit, though whether this significantly impacts overall enterohepatic efficiency or primarily represents a localized phenomenon requires further investigation [34]. ET-R typically demonstrates moderate fecal BA excretion (approximately 4–6% of pool) and intermediate turnover rates, with the unique contribution of iso-bile acids to metabolic signaling and pool homeostasis remaining an active area of research [26,38].

These enterotype-specific functional differences highlight how gut microbial composition tunes BA homeostasis through differential transformation capacity, with direct implications for hepatic metabolism, intestinal nutrient absorption, nuclear receptor signaling, and systemic metabolic regulation. The recognition that enterotypes establish distinct BA “metabolotypes” has important implications for understanding inter-individual variation in responses to dietary interventions, BA-based therapeutics, and metabolic disease susceptibility.

### 3.3. BA Signaling: The FXR and TGR5 Pathway

BAs function as master metabolic regulators through two primary receptors—the nuclear FXR and the membrane-bound G-protein-coupled bile acid receptor 1 (TGR5/GPBAR1)—which translate variations in BA pool composition into systemic metabolic signals [66]. FXR exhibits differential binding affinities among BAs, with CDCA being the most potent, followed by DCA and CA, while LCA is a relatively weak FXR agonist [67]. Hepatic FXR activation induces expressions of small heterodimer partner (SHP) and fibroblast growth factor 19 (FGF19), which subsequently suppresses cholesterol 7α-hydroxylase (*CYP7A1*), the rate-limiting enzyme in BA synthesis, thereby preventing BA overproduction and hepatotoxic accumulation [68]. FXR also upregulates the bile salt export pump (BSEP), integrates lipid and glucose metabolism, and contributes to anti-inflammatory regulation [5,69]. In the intestine, FXR activation coordinates gut–liver communication by modulating FGF19 secretion, incretin hormone release, and hepatic gluconeogenic control [70].

TGR5, in contrast to FXR, preferentially responds to secondary BAs, with LCA and DCA exhibiting the highest agonist potency, making TGR5 activation strongly enterotype-dependent [51,71]. TGR5 stimulation increases energy expenditure through type 2 iodothyronine deiodinase (DIO2)-mediated thyroid hormone activation, enhances glucagon-like peptide (GLP)-1 release to improve glucose homeostasis, and modulates macrophage polarization toward anti-inflammatory phenotypes [72]. Consequently, ET-B, characterized by high DCA and LCA production, promotes stronger TGR5 activation and greater energy expenditure compared to ET-P, which maintains predominantly primary BAs with lower TGR5 agonist activity. The differential TGR5 activation across enterotypes contributes to individual variability in metabolic efficiency and glycemic control [73].

The integrated FXR–TGR5 network represents a finely tuned communication system linking microbial BA metabolism with host energy balance, inflammation, and immune regulation [74]. The enterotype-dependent BA composition creates distinct receptor activation profiles: ET-B’s secondary BA-enriched pool favors TGR5 activation while maintaining moderate FXR signaling; ET-P’s primary BA predominance provides strong CDCA-mediated FXR activation with reduced TGR5 engagement; and ET-R’s unique iso-BA production may modulate both pathways through structurally modified ligands with altered receptor binding properties. Individuals may exhibit distinct metabolic responses to identical dietary or pharmacologic interventions because the enterotype-specific composition of BAs determines receptor activation. Understanding these enterotype-driven signaling pathways provides a framework for precision medicine that targets BA-mediated metabolic regulation and host–microbiome co-adaptation [66,75].

## 4. Microbial Transformation of BAs Across Enterotypes

### 4.1. Enzymatic Machinery: BSH as the Deconjugation Gateway

BA transformation in the gut is initiated by a complex network of microbial enzymes (Table 1), among which BSH plays the most fundamental and widespread role. These enzymes catalyze the hydrolysis of the amide bond linking primary BAs to conjugated amino acids (glycine or taurine), generating unconjugated BAs and free amino acids [74]. This deconjugation step serves as the obligatory gateway for all subsequent BA biotransformations, positioning the BSH activity as the primary determinant of the BA metabolic potential of a microbial community [3]. BSH genes are ubiquitous across diverse bacterial taxa, including *Bacteroides*, *Lactobacillus*, *Bifidobacterium*, *Enterococcus*, and *Clostridium*, with many species harboring multiple paralogs that exhibit distinct substrate preferences and regulatory controls [76]. Even archaeal species such as *Methanobrevibacter smithii* encode functional BSH homologs, indicating evolutionary conservation of function [77]. This functional redundancy across diverse taxa provides metabolic resilience, ensuring that robust BA processing capacity is maintained despite shifting in microbial community composition.

BSHs differ markedly in substrate specificity and biochemical properties [77]. Some isoforms preferentially hydrolyze glycine-conjugated BAs, whereas others display a higher affinity for taurine-conjugated forms [77]. Such substrate selectivity creates functional complementarity within microbial consortia, enabling complete deconjugation of the BA pool entering the colon. The liberated unconjugated BAs serve as substrates for downstream microbial enzymes, linking BSH activity directly to the formation of bioactive secondary BAs [83].

### 4.2. Enzymatic Machinery: 7α-Dehydroxylases and Secondary BA Formation

Following deconjugation, the 7α-dehydroxylation pathway is the most biochemically complex and functionally significant microbial transformation of BAs [3]. This multi-step process removes the C7 hydroxyl group from CA and CDCA to generate the secondary BAs, DCA, and LCA, respectively. These transformations substantially alter the hydrophobicity of BAs, their membrane affinity, and receptor activation profiles, producing molecules with distinct biological and signaling properties [3]. The 7α-dehydroxylation process requires coordinated redox reactions and specialized cofactors, restricting its activity to a narrow subset of anaerobic gut bacteria capable of sustaining these energy-demanding reactions. *Clostridium scindens* is the prototypical 7α-dehydroxylating bacterium, although homologous genes have also been detected in other *Clostridiales* species, including members of the *Lachnospiraceae* and *Ruminococcaceae* families [79]. The abundance and activity of these bacteria largely dictate the capacity for secondary BA production—an essential determinant of host metabolic signaling due to the potent receptor affinities of DCA and LCA for TGR5 and FXR [83].

### 4.3. Genetic Basis: The Bai Operon—Blueprint for BA Dehydroxylation

The BA-inducible (*bai*) operon represents one of the most striking examples of bacterial metabolic specialization [3]. This coordinated gene cluster encodes the complete enzymatic and regulatory machinery required for 7α-dehydroxylation. Typically comprising eight to ten genes (*baiB*, *baiCD*, *baiE*, *baiA*, *baiF*, *baiG*, *baiH*, *baiI*), the operon includes enzymes catalyzing oxidation–reduction reactions, electron transport proteins, and transcriptional regulators to ensure BA-responsive expression [3].

Transcriptional activation of the *bai* operon occurs in response to elevated luminal concentrations of BAs—particularly CDCA and CA—allowing bacteria to economize energy by inducing this pathway only when substrates are abundant [78]. Within the operon, *baiA* encodes a 7α-dehydratase initiating oxidation, while *baiB* and *baiE* function as reductases completing the conversion to secondary BAs. Other genes contribute to electron transport (*baiCD*, *baiG*, *baiH*) and cofactor regeneration (*baiF*, *baiI*), creating a fully integrated biochemical system [78]. Functional coupling between BSH and *bai* operon activities exemplifies sequential microbial cooperation in BA metabolism. BSH-positive bacteria first liberate unconjugated BAs, which then serve as substrates for 7α-dehydroxylating species harboring the *bai* operon [78]. This syntrophic relationship not only facilitates BA turnover but also confers ecological advantages—secondary BAs exhibit antimicrobial properties that shape community composition and maintain ecosystem stability.

### 4.4. Enterotype-Specific Enzymatic Profiles and Metabolic Capacity

Distribution and activity of enzymes that transform BAs vary systematically across enterotypes, producing distinct metabolic signatures that influence both BA composition and host physiology (Table 2) [46]. ET-B Profile: *ET-B* exhibits the highest overall BSH activity due to extensive BSH gene repertoires in *B. uniformis*, *B. ovatus*, and *B. fragilis*. These species collectively encode dozens of isoenzymes with complementary substrate specificities, supporting the near-complete deconjugation of glycine- and taurine-conjugated BAs. ET-B communities also harbor moderate 7α-dehydroxylating activity from associated *Clostridiales*, producing elevated levels of secondary BAs, particularly DCA, and demonstrating strong potential to activate FXR/TGR5 [84,85].

ET-P Profile: ET-P displays moderate BSH activity and *Prevotella* spp. harbor fewer BA transformation genes compared to ET-B. Their reduced 7α-dehydroxylase activity results in a primary BA-enriched profile with lower hydrophobicity. Functional adaptations in ET-P prioritize complex carbohydrate fermentation (leading to high SCFA production) and the clearance of less-transformed BAs, reflecting distinct ecological and metabolic strategies [23,24,26].

ET-R Profile: *ET-R* demonstrates intermediate BSH activity and variable 7α-dehydroxylation capacity. Species such as *R. bromii* possess unique carbohydrate-binding domains that facilitate BA sequestration on the cell surface polysaccharides, modulating BA availability and reabsorption kinetics. These bacteria often coexist with moderate secondary BA producers, generating balanced BA pools that support host metabolic stability [14,34,86].

Enterotype-specific enzymatic constellations are reinforced by BA–microbiome feedback loops. Distinct BA environments selectively favor bacteria with compatible metabolic repertoires, stabilizing community structures over time despite dietary fluctuations. Understanding these functional networks provides a crucial insight into interindividual variability in BA signaling and therapeutic responsiveness to BA-modifying interventions [46,87].

## 5. The Host Genetic Determinants of BA-Enterotype Interactions

### 5.1. BA Synthesis and Transport Gene Polymorphisms

**Figure 2:**
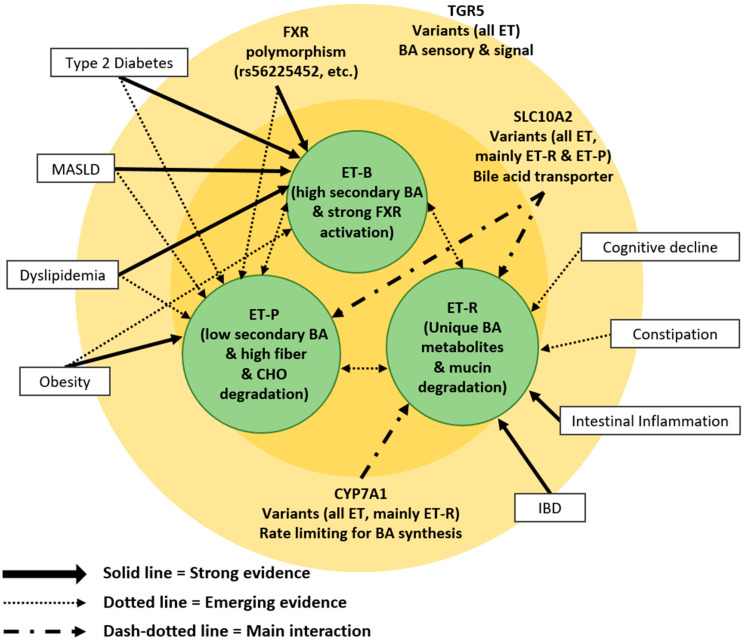
Interplay of Host Genetics and Gut Enterotypes in Bile Acid-Mediated Disease Susceptibility.

Host genetic variations in BA synthesis, conjugation, and transport pathways play a decisive role in shaping enterotype composition and stability by modulating the biochemical environment that selects for specific microbial communities [88] (Table 3), resulting in substrate-limited luminal environments that favor enterotypes with enhanced BA conservation and detoxification mechanisms, particularly ET-P communities [89]. Conversely, higher CYP7A1 activity generates BA-rich luminal conditions that promote ET-B, characterized by robust BSH activity and extensive secondary BA transformation capacity [90]. Polymorphisms in *CYP8B1*, which encodes sterol 12α-hydroxylase, further shape enterotype distribution by altering the ratio of CA and CDCA in the primary BA pool. The *CYP8B1* rs3732860 variant reduces 12α-hydroxylation efficiency, leading to CDCA-dominant BA profiles that favor microbial communities adept at CDCA metabolism—typically those associated with ET-P (Figure 2) [91].

In addition, host variation in BA conjugation enzymes such as BACS and BAAT modifies the conjugation ratio with glycine and taurine in primary BAs. Since bacterial BSHs display distinct substrate preferences, this compositional shift directly alters the microbial transformation patterns [92]. Variants in ileal transport and reuptake proteins—OST-α/β (SLC51A/B) and IBABP/FABP6—modulate enterohepatic recirculation efficiency, thereby controlling colonic exposure to BAs. Reduced transport activity increases substrate availability for microbial metabolism, strengthening the feedback between the host genotype and the enterotype structure [93,94].

**Table 3:** Key Host Transporters and Receptors in Bile Acid (BA) Homeostasis.

Transporter/Receptor	Location	Primary Function	Associated Pathway	Study Type; REF
Bile Salt Export Pump (*BSEP*, *ABCB11*)	Hepatocyte Canalicular Membrane	Major efflux system for BAs into bile canaliculi.	Enterohepatic Circulation	in vitro, in vivo; [95]
Sodium Taurocholate Cotransporting Polypeptide (*NTCP*, *SLC10A1*)	Hepatocyte Basolateral Membrane	Active uptake of conjugated BAs from portal blood into hepatocytes.	Enterohepatic Circulation	human (cross-sectional); [96]
Farnesoid X Receptor (*FXR*)	Intestine, Liver (Nuclear Receptor)	Endogenous ligand-activated receptor; regulates BA synthesis and lipid/glucose metabolism.	Negative Feedback Loop, Host Signaling	in vitro, in vivo; [97]
G Protein-Coupled BA Receptor 1 (*TGR5*)	Intestine, Immune Cells (Membrane Receptor)	Endogenous ligand-activated receptor; modulates energy, glucose, and inflammation.	Host Signaling	in vitro, in vivo; [6]
Apical Sodium-Dependent BA Transporter (*ASBT*)	Terminal Ileum	Active reabsorption of BAs from the intestinal lumen.	Enterohepatic Circulation)	in vitro, in vivo; [98]

REF: reference.

### 5.2. Nuclear Receptor Gene Polymorphisms: FXR and TGR5 Signaling

Genetic variations in nuclear and membrane-bound BA receptors create individual-specific response patterns to microbiota-derived BA signals, establishing personalized metabolic feedback loops [6,99]. The *FXR* variant rs56163822 in the ligand-binding domain modifies receptor affinity for secondary BAs—especially DCA and LCA, which are enriched in ET-B environments [100]. Carriers display altered dose–response curves and attenuated feedback inhibition of hepatic BA synthesis, potentially predisposing to dysregulated BA pools and distinct metabolic phenotypes. The same variant in the *FXR* promoter region influences tissue-specific expression, leading to variable hepatic versus intestinal FXR activity and thereby modulates BA-mediated regulation of glucose and lipid metabolism [101].

The *TGR5* receptor variant rs11554825 alters receptor expression in metabolically active tissues, including brown adipose tissue and enteroendocrine cells. This polymorphism affects thermogenic capacity, glucagon-like peptide-1 (GLP-1) secretion, and postprandial glucose regulation in response to secondary BA exposure [102,103]. As TGR5 exhibits its highest affinity for DCA and LCA, this genetic variability amplifies the metabolic divergence observed between enterotypes dominated by secondary BA producers (ET-B) and those with reduced dehydroxylation activity (ET-P). Together, the *FXR* and *TGR5* polymorphisms fine-tune BA signaling intensity and cross-tissue integration, reinforcing how host genetics and microbial metabolism jointly define systemic metabolic outcomes [104].

### 5.3. SLC10A2 Polymorphisms and Enterotype-Specific BA Availability

The *SLC10A2* gene, encoding the apical ASBT/ileal BA transporter (IBAT), exerts a direct and profound control over BA substrate availability for colonic microbial metabolism. Functionally significant nonsynonymous polymorphisms—*292G>A (p.Ala98Thr)* and *431G>A (p.Asp144Asn)*—reduce ASBT transport efficiency and taurocholate uptake [105]. Individuals carrying these variants exhibit increased fecal BA loss and compensatory upregulation of hepatic synthesis, producing luminal environments enriched in BA substrates that strongly favor ET-B communities with high BSH and 7α-dehydroxylation capacity. These genotype-driven differences extend beyond microbiome composition to influence therapeutic and dietary responses. Reduced ASBT function alters individual responses to BA sequestrants and may modify adaptation to high-fat diets by reshaping EHC dynamics [106].

Quantitative trait locus (QTL) analyses link *SLC10A2* variants with both *Turicibacter* abundance and plasma cholic acid concentrations, suggesting shared genetic control over microbial and host BA traits [88]. Moreover, population-level differences in *SLC10A2* allele frequencies correlate with dietary adaptation: variants promoting BA conservation are enriched in populations consuming high-fiber diets, whereas variants supporting higher turnover rates are more prevalent in populations historically exposed to animal-based diets [107]. This evolutionary divergence underscores the co-adaptation of host genetics, diet, and microbiome ecology.

### 5.4. Gene–Environment–Microbiome Interactions

Host genetic effects on BA metabolism are profoundly modulated by environmental and pharmacological factors that shape BA signaling and microbial ecology. The nuclear BA receptor FXR integrates with cellular energy pathways through interactions with adenosine monophosphate-activated protein kinase (AMPK), which phosphorylates and attenuates FXR activity [105]. Pharmacological modulation illustrates this complexity. Metformin, a first-line antidiabetic agent, interferes with BA homeostasis via AMPK–FXR crosstalk, reducing FXR-mediated transcription of the genes governing BA conjugation, uptake, and export [108]. Such medication-induced alterations can override or amplify genetic predispositions, influencing both baseline enterotype composition and its dynamic transitions under environmental stressors. Individuals with BA conservation-promoting variants, such as reduced cholesterol 7α-hydroxylase or ASBT function, tend to exhibit greater enterotype stability, while those with BA turnover-facilitating alleles display more adaptive microbial responses to dietary fat or pharmacologic interventions [109].

Functional experiments further illustrate the physiological implications of these interactions. Mice lacking the *CYP8B1* gene—encoding the enzyme required for 12α-hydroxylated BA synthesis—show resistance to Western diet-induced obesity and hepatic steatosis, largely due to impaired intestinal fat absorption [110]. These effects are reversible with dietary fat restriction, demonstrating how BA composition regulates systemic energy homeostasis. Similarly, inhibition of ASBT (encoded by *SLC10A2*) improves metabolic and intestinal inflammation profiles in animal models [111], highlighting the therapeutic potential of targeting the BA–microbiome–host axis.

Collectively, these findings emphasize the triadic interplay between host genetics, environment, and microbiome, wherein genetic determinants establish baseline BA signatures, environmental exposures reshape regulatory networks, and microbial communities translate these signals into metabolic outcomes [11]. Future integrative research combining genomics, metabolomics, and microbiome profiling will be essential for developing personalized strategies for managing metabolic and gastrointestinal diseases through the targeted modulation of BA signaling.

## 6. Enterotype-Specific BA Profiles and Disease Signatures

### 6.1. Hepatic Diseases: MASLD/MASH and Cirrhosis

Enterotypes exhibit distinctive BA metabolic profiles that translate into specific disease susceptibilities (Table 4; Figure 2). MASLD and its progressive form, MASH, demonstrate profound enterotype-dependent variations in BA metabolism that shape disease trajectory [112]. Patients with MASH consistently present with elevated total fecal BAs and increased primary-to-secondary BA ratios, reflecting impaired microbial transformation capacity due to the reduced abundance of 7α-dehydroxylating species such as *Clostridium leptum* [113]. Enterotype-specific associations reveal clinically meaningful patterns: ET-B predominates in early-stage MASLD, whereas ET-P shows a markedly higher risk of progression to cirrhosis—approximately a 33% increase compared with ET-B [22,114]. This gradient suggests that the progression of liver diseases may be stratified by underlying enterotype configuration. The ET-P community, characterized by reduced secondary BA metabolism and diminished SCFA production, may foster pro-inflammatory and fibrinogenic environments that accelerate hepatic injury.

The role of *Prevotella* exemplifies the functional heterogeneity within enterotypes. While some strains enhance host metabolic health through SCFA production (notably propionate), others produce virulence factors and endotoxins promoting the inflammatory milieu characteristic of MASH [33,117]. The high-risk ET-P2 subtype shows a nearly two-fold increased risk of obesity and diabetes [118]. In advanced cirrhosis, reduced hepatic BA synthesis leads to substrate depletion, causing the collapse of secondary BA-producing bacterial populations and lowering fecal DCA/CDCA and LCA/CA ratios [112]. This disruption perpetuates a deleterious feedback loop—hepatic dysfunction drives dysbiosis, while microbial imbalance exacerbates liver injury through impaired BA signaling and compromised intestinal barrier integrity [118].

### 6.2. Metabolic Disorders: Type 2 Diabetes

ET-B is strongly associated with obesity and metabolic dysregulation through the enhanced energy harvest from the dietary proteins and fats typical of Western diets [81]. These communities, particularly ET-B2, characterized by low α-diversity, produce elevated levels of branched-chain amino acids and trimethylamine N-oxide (TMAO) precursors, alongside BA signatures enriched in secondary species such as DCA, which activate pro-inflammatory signaling pathways. In T2D, BA alterations exhibit clear enterotype specificity [119]. Diabetic individuals display increased circulating DCA levels, primarily derived from enhanced microbial transformation by *Clostridium scindens*, disrupting glucose homeostasis through aberrant FXR and TGR5 signaling [119,120]. Elevated secondary BA activity contributes to impaired insulin sensitivity and increased hepatic glucose output. The frequent association between *Lactobacillus* abundance and diabetes may partly reflect the effects of medication rather than direct causation. Metformin, for instance, modifies the gut microbial composition and BA metabolism through AMPK–FXR crosstalk, underscoring the need for functional (metabolite-based) rather than purely taxonomic interpretation. ET-P demonstrates context-dependent metabolic effects: while high-fiber diets support metabolic health through SCFA production, specific ET-P2 configurations are paradoxically associated with a higher risk of obesity and diabetes [16].

### 6.3. Inflammatory Bowel Disease (IBD): Intestinal Integrity and Immune Modulation

IBD exemplifies how BA dysregulation within distinct enterotypes contributes directly to inflammatory pathogenesis. Patients exhibit characteristic BA signatures—elevated conjugated primary BAs such as glycocholic acid and reduced secondary BAs (DCA, LCA)—reflecting the loss of the *bai* operon-carrying bacteria responsible for secondary BA formation [121].

ET-P is particularly linked with chronic intestinal inflammation through two primary mechanisms: compromised epithelial barrier integrity and dysregulated immune signaling. Loss of secondary BAs eliminates TGR5-mediated anti-inflammatory signaling in intestinal macrophages, reducing phosphorylation of cyclic AMP-response element-binding protein (CREB) and permitting overproduction of tumor-necrosis factor (TNF)-α and interleukin (IL)-1β while diminishing IL-10 [85,121,122,123,124]. Consequently, persistent inflammation results from the absence of secondary BA-derived immunomodulatory feedback [125]. This mechanistic connection highlights the causal role of microbial BA metabolism in intestinal immune homeostasis and IBD pathophysiology.

### 6.4. Colorectal Cancer: Secondary BAs and Carcinogenesis Risk

The interplay between enterotype structure, BA metabolism, and colorectal cancer (CRC) risk illustrates how microbial communities influence carcinogenesis through metabolic modulation. Secondary BAs—particularly DCA and LCA—are potent tumor-promoting agents [126,127]. DCA induces genomic instability via reactive oxygen species-mediated DNA damage, while LCA suppresses the DNA repair mechanisms. Both hydrophobic BAs enhance epithelial proliferation, inhibit apoptosis, and promote activation of oncogenic pathways, including epidermal growth factor receptor (EGFR)-mitogen-activated protein kinase (MAPK), phosphoinositide 3-kinase (PI3K)-AKT, and nuclear factor-kappa B subunit 1 (NF-κB) signaling [87,128,129].

ET-B, commonly associated with Western dietary habits, elevates concentrations of secondary BAs and is correspondingly linked with higher CRC risk [126]. The risk is compounded by the decreased abundance of butyrate-producing bacteria such as *Faecalibacterium* and *Roseburia*, whose SCFA production normally protects colonic epithelial integrity [130,131].

### 6.5. Predictive Biomarkers and Disease Progression Patterns

Integrating enterotype classification with BA profiling provides a robust framework for disease risk assessment and precision medicine [39]. Quantitative correlations between microbial abundance and specific BA concentrations enable the identification of taxa actively engaged in BA transformations [132]. The serum primary-to-secondary BA ratio has emerged as a validated biomarker for assessing disease activity in IBD and other disorders with BA dysregulation [112]. However, clinical application requires a nuanced understanding. Enterotypes represent ecological tendencies rather than fixed categories, and substantial functional heterogeneity exists even within the same enterotype. Interactions with diet, host genetics, and pharmacologic exposures further modulate BA dynamics. Thus, functional metabolic capacity, particularly the ability for BA deconjugation, 7α-dehydroxylation, and re-conjugation, should be prioritized over static taxonomic definitions.

Future therapeutic approaches will probably target the enterotype–BA axes to restore metabolic homeostasis. Such strategies may include precision probiotics, BA receptor modulators, or diet-based interventions designed to optimize enterotype configuration, normalize BA signaling, and ultimately improve clinical outcomes.

## 7. Enterotype-Guided Therapeutic Strategies

### 7.1. Precision Nutrition and Dietary Interventions

Recognition that distinct enterotypes respond differentially to identical dietary interventions supports a shift toward enterotype-stratified precision nutrition as an intermediate and pragmatic step toward fully individualized dietary strategies. Unlike population-averaged recommendations, this approach acknowledges that dietary metabolic outcomes are mediated by each microbiome’s unique BA-transforming capacity, which varies systematically across enterotypes. Furthermore, functional heterogeneity within major enterotypes necessitates finer stratification, as metabolic subtypes display divergent BA signatures and disease associations (Table 4).

Within ET-B, the ET-B2 subtype—dominated by *B. thetaiotaomicron* and related species—exhibits high bile salt hydrolase and hydroxysteroid dehydrogenase activity, leading to extensive BA deconjugation and secondary modification. These features correlate with increased risks of obesity, hypertension, and metabolic disease [81,133]. In contrast, the ET-P2 subtype demonstrates elevated enzymatic capacity for lithocholic acid (LCA) metabolism, producing high LCA levels linked to obesity, diabetes, and intestinal inflammation [48]. In IBD, characteristic microbial configurations demonstrate reduced diversity of BA-modifying bacteria with diminished *bsh* and *bai* operon activity, resulting in the accumulation of primary BAs and loss of secondary BAs—an imbalance that contributes to mucosal inflammation and barrier dysfunction [134]. Emerging enterotype categories extend beyond the classic *Bacteroides* and *Prevotella* dichotomy. *Enterobacteriaceae*-enriched profiles have been observed in irritable bowel syndrome, while *Bifidobacterium*-dominated communities enhance BA hydrogel formation and improve mucosal protection in gastrointestinal disorders [116]. These refined subtypes underscore the need for enterotype classification systems that integrate both compositional and functional metabolic capacity to guide individualized dietary strategies.

Individuals with ET-P typically exhibit superior metabolic responses to high-fiber diets due to their robust capacity for complex carbohydrate fermentation and SCFA production [135,136]. Clinical evidence shows that fiber-rich diets elicit greater weight loss, improved glucose homeostasis, and reduced cardiovascular risk in ET-P individuals than in *Bacteroides*-dominant counterparts. Mechanistically, *Prevotella* communities ferment dietary fiber into butyrate, propionate, and acetate, enhancing colonic health while binding BAs and modulating enterohepatic recirculation [77,137].

Conversely, ET-B individuals benefit from dietary approaches that moderate excessive secondary BA production while optimizing protein and lipid metabolism [8]. Strategies may include selective inclusion of BA-binding fibers such as psyllium and oat β-glucan to attenuate hydrophobic BA exposure, alongside prebiotic compounds that favor beneficial *Bacteroides* strains [138]. Fiber type is particularly relevant: soluble fibers enhance SCFA production in ET-P, whereas insoluble fibers more effectively bind and remove BAs in ET-B [8]. Additionally, circadian rhythm-based meal timing and macronutrient distribution may require enterotype-specific optimization, as both BA synthesis and microbial metabolism demonstrate circadian regulation.

### 7.2. Microbiome-Targeted Therapies

Direct modulation of gut microbial communities provides precise leverage points for correcting BA imbalances across enterotypes. Probiotic and prebiotic interventions should prioritize strains with characterized BA-transforming functions rather than broad-spectrum effects [4,139]. *Lactobacillus* and *Bifidobacterium* species with defined bile salt hydrolase activity can strategically alter BA deconjugation and cholesterol metabolism. However, therapeutic efficacy depends strongly on the host’s baseline enterotype: selective pressures differ markedly between *Bacteroides*- and *Prevotella*-dominated ecosystems, requiring tailored formulations to achieve desired metabolic effects [140,141].

Fecal microbiota transplantation (FMT) represents the most comprehensive microbiome restoration approach, with demonstrated efficacy in reestablishing BA-transforming functions in dysbiotic conditions [142]. In IBD, FMT restores secondary BA-producing bacteria, normalizing serum 7α-hydroxy-4-cholesten-3-one levels and alleviating clinical symptoms [140]. Advanced microbiome-directed therapeutics are evolving toward defined microbial consortia—engineered bacterial communities designed to reconstitute BA metabolism with predictable outcomes [142]. Such synthetic consortia can be tailored to individual enterotypes, representing a major advance over traditional probiotic approaches.

### 7.3. Pharmacological Modulation of BA Signaling

Dietary and pharmacological targeting of BA receptors provides a complementary strategy to dietary and microbiome-based interventions [143]. FXR agonists improve BA homeostasis, glucose regulation, and lipid metabolism, yet their therapeutic efficacy and safety windows vary by enterotype due to baseline differences in FXR activation [6,120]. Individuals with *Bacteroides*-dominant microbiota, typically exhibiting high secondary BA levels, may require lower FXR agonist doses compared to those with *ET-P* [144]. Gut-restricted FXR agonists such as fexaramine are particularly suited for enterotype-specific therapy, exerting localized intestinal effects while minimizing systemic exposure [145]. TGR5 agonists offer additional benefits for enterotypes characterized by reduced secondary BA production, enhancing energy expenditure, glucose tolerance, and anti-inflammatory signaling [45]. BA sequestrants provide another class of agents that alter EHC efficiency, though their clinical impact also varies across enterotypes. The next generation of BA therapeutics—engineered derivatives with receptor selectivity and optimized pharmacokinetics—promises finer modulation of BA signaling across diverse metabolic contexts.

### 7.4. Enterotype-Specific Integration Strategies

The intricate interplay between BAs, microbial ecology, and host metabolism necessitates integrated therapeutic frameworks combining dietary, microbiological, and pharmacological modalities tailored to enterotype-specific characteristics (Table 4). For ET-B, the primary goal is to mitigate excessive secondary BA accumulation, particularly DCA and LCA, which drive metabolic inflammation and colorectal carcinogenesis [146]. Effective interventions combine moderate-fat dietary patterns with BA-binding fibers to sequester toxic hydrophobic BAs. Adjunctive strategies include BSH-active probiotics and antioxidant-rich vegetables to counteract oxidative stress induced by secondary BAs [147]. If additional modulation is required, selective FXR or TGR5 agonists can be used to rebalance BA signaling in this high-secondary-BA environment.

For ET-P individuals, particularly those in Asian and African populations consuming high-carbohydrate, low-protein diets, therapeutic strategies focus on correcting macronutrient imbalances [2]. Increasing lean protein and fish intake while reducing refined carbohydrates suppresses *P. copri* overgrowth and limits LCA accumulation [148]. Supportive interventions may include probiotics, enhancing SCFA production, and pharmacologic agents targeting altered BA signaling during microbiome transition. The goal is not to sustain *Prevotella* dominance but to achieve a stable, metabolically favorable configuration through nutritional recalibration.

ET-R, often associated with constipation and mucin degradation, requires specialized intervention [149]. Regular consumption of fermented foods such as kimchi, sauerkraut, and kefir enhances motility and microbial diversity [150]. Nutrients supporting mucosal defense, with omega-3 fatty acids, zinc, and vitamin A, maintain barrier integrity despite mucin degradation by *R. torques* [151]. Adequate hydration, soluble fibers, and prebiotics such as inulin and fructo-oligosaccharides further promote beneficial commensals and counterbalance mucin degraders. Together, these strategies address both impaired transit and BA dysregulation linked to mucosal compromise.

### 7.5. Implementation Considerations and Advanced Approaches

Temporal coordination of interventions is crucial, as BA metabolism and microbial activity follow circadian patterns [152]. Morning administration of BA sequestrants in ET-B may enhance binding during peak hepatic synthesis, while timing resistant starch intake to late-day periods may optimize fermentation dynamics in dysbiotic states. Probiotic administration should align with meal timing and nutrient composition to facilitate colonization and metabolic integration [153]. Personalized monitoring is indispensable. Longitudinal tracking of enterotype stability, BA profiles (serum 7α-hydroxy-4-cholesten-3-one, total BAs, ratio of primary and secondary BAs), metabolic biomarkers (glucose, lipids, inflammation), and clinical endpoints allows dynamic optimization of treatment regimens. Emerging point-of-care technologies for rapid BA quantification and metagenomic enterotyping will soon enable real-time therapeutic adjustment based on objective metabolic feedback rather than subjective symptom relief.

Synthetic biology is poised to revolutionize this field. Engineered bacterial strains can be programmed to express specific BA-modifying enzymes—BSH, hydroxysteroid dehydrogenases, or bai operon components—customized to individual therapeutic needs. Such biotherapeutics could, for example, suppress excessive secondary BA production in ET-B or enhance butyrate synthesis while minimizing mucin degradation in ET-R. Integrating these innovations with traditional nutrition and pharmacology will allow unprecedented precision in managing metabolic and inflammatory disorders.

Safety of microbiota-targeted therapy remains unclear. Combination regimens should consider potential nutrient–microbe–drug interactions and avoid excessive BA depletion, which can impair fat-soluble vitamin absorption or disrupt FXR-mediated regulation [154]. Excessive or poorly targeted microbiome interventions, such as repeated fecal microbiota transplantation or high-dose probiotic supplementation, may disrupt established microbial community stability. A phased, closely monitored approach—combining mechanistic insight with clinical vigilance—ensures therapeutic efficacy while minimizing adverse effects [155]. Ultimately, the integration of enterotype-specific strategies (Table 5) provides a clinically actionable framework for personalized medicine rooted in BA–microbiome–host crosstalk. As understanding of enterotype subtypes, genetic modifiers, and environmental determinants advances, these foundational principles will continue to evolve, enabling increasingly precise and effective interventions for metabolic, hepatic, and intestinal diseases.

## 8. Clinical Implementation and Future Outlook

### 8.1. From Research to Practice: Challenges and Opportunities

**Figure 3:**
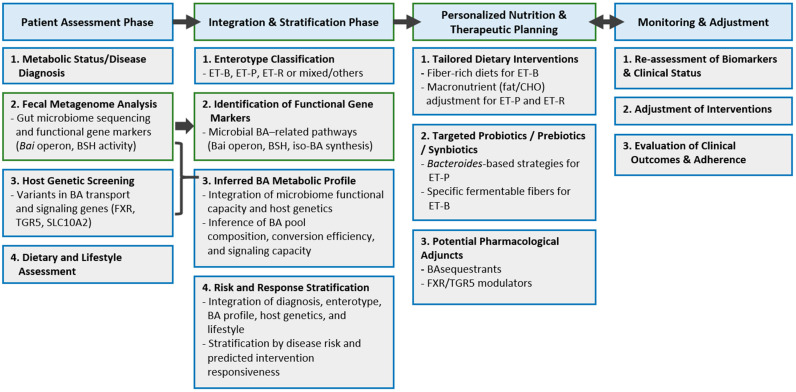
Precision Nutrition Clinical Workflow: Integrating Microbiome, Bile Acid Metabolism, and Host Genomics.

Translating enterotype-based precision nutrition into clinical practice faces substantial scientific and logistical challenges. The human gut microbiome exhibits considerable inter-individual and temporal variability, complicating the establishment of reproducible therapeutic frameworks (Figure 3). Current evidence suggests that 30–40% of individuals fail to respond to standard microbiome-based interventions, underscoring the need for more precise, mechanistically informed strategies [15,156]. Practical barriers remain substantial: The absence of consensus protocols for enterotype classification, limited clinical access to high-throughput microbiome sequencing, and uncertainty about cost-effectiveness hinder widespread adoption [157]. These limitations must be addressed through harmonized diagnostic standards, simplified testing pipelines, and demonstration of clear clinical benefit to support healthcare integration and reimbursement.

### 8.2. Essential Biomarkers for Guiding Precision Therapies

Clinically actionable biomarkers are pivotal for transforming enterotype research into therapeutic applications. Moving beyond purely taxonomic frameworks, functional biomarkers that quantify BA metabolic capacity offer greater translational potential. Among these, serum 7α-hydroxy-4-cholesten-3-one has emerged as a reliable indicator of hepatic BA synthesis and microbial transformation efficiency, identifying individuals most responsive to BA-modulating interventions such as FMT [140]. Complementary markers—such as fecal BA composition, primary/secondary BA ratios, and microbial gene signatures (e.g., bai operon, BSH activity)—can provide real-time insights into gut functional status, enabling therapy calibration without extensive metagenomic sequencing [140].

### 8.3. The Path Forward: Clinical Validation and Advanced Analytics

Future advancement depends on large, prospective trials validating the superiority of enterotype-informed interventions over conventional dietary recommendations. Integration of multi-omic datasets through machine learning and network modeling will enable the prediction of individual therapeutic responses by linking microbial composition, host genetics, and BA dynamics [156,158]. Continued discovery of uncharacterized microbial taxa, novel BA-related genes, and secondary metabolites will expand the therapeutic toolkit. Establishing standardized microbiome diagnostic pipelines, rigorous quality control, and appropriate regulatory frameworks is essential for clinical translation [159]. Ultimately, combining enterotype-based strategies with other precision medicine modalities—including pharmacogenomics and personalized metabolomics—will enhance predictive accuracy and therapeutic efficacy, propelling microbiome-guided precision health toward routine clinical reality.

### 8.4. Limitations

This review has several limitations that reflect the current state of the evidence base. First, the interpretation of enterotypes remains constrained by the lack of standardized classification methods, leading to variability across studies in how community types are defined and labeled. This heterogeneity limited direct comparison across datasets and required cautious interpretation of functional patterns. Second, available studies differ widely in sequencing platforms, depth, BA quantification techniques, and dietary assessment methods, making it difficult to draw fully harmonized conclusions across research populations. Third, although both human and mechanistic animal studies were included, the evidence remains uneven: many mechanistic insights come from animal models, whereas long-term human intervention data linking enterotypes, BA metabolism, and dietary responses are still limited. Fourth, as a narrative review, study selection may be subject to selection bias despite an effort to search major databases systematically. These constraints may affect the strength of causal inferences and highlight areas where current conclusions should be viewed as provisional pending further validation.

### 8.5. Future Research Directions

To address these limitations, future research should prioritize large, longitudinal human studies that directly test whether enterotype-informed dietary interventions outperform standard population-based recommendations. Standardized analytical pipelines for enterotype assignment, microbial gene quantification (*BSH* and *bai* gene clusters), and fecal or serum BA profiling are essential to improve reproducibility and facilitate cross-cohort comparisons. Multi-omic integration—including metagenomics, metabolomics, host genetics, and dietary intake—combined with advanced analytical approaches such as machine learning, will be necessary to predict individual therapeutic responses with greater accuracy. Additionally, further discovery and functional validation of uncharacterized microbial taxa and BA-modifying genes will help refine mechanistic models. Ultimately, clinical translation will require reproducible diagnostic frameworks, clear clinical utility, and cost-effective testing strategies to make enterotype-informed precision nutrition feasible in practice.

## 9. Conclusions

The enterotype–BA framework offers a powerful lens for interpreting inter-individual variability in diet–microbiome–host interactions. Enterotype-associated community patterns—often enriched in *Bacteroides*, *Prevotella*, or *Ruminococcus*—generate distinct BA metabolic signatures that modulate FXR and TGR5 signaling and shape metabolic, inflammatory, and cardiometabolic outcomes. Understanding these functionally divergent microbial configurations helps explain why individuals respond differently to identical dietary inputs and supports the rationale for enterotype-stratified precision nutrition. Recent advances in functional microbiome biomarkers, synthetic biology, and computational modeling are making individualized modulation of BA metabolism increasingly feasible. However, standardization of enterotype definitions, longitudinal stability assessment, and clinical validation remain essential next steps. Integrating enterotype-informed nutrition, microbiome-directed therapies, and BA-targeted pharmacology represents a promising path toward microbiome-based precision medicine—one that aims not only to treat disease, but to optimize metabolic health through alignment with each individual’s microbial ecology.

## Figures and Tables

**Table 1:** Microbial Enzymes and Their Bile Acid Biotransformations.

Enzyme/Operon	Microbial Source (Genus)	Catalyzed Reaction	Resulting BA Product	REF
Bile Salt Hydrolase (*BSH*)	*Bacteroides*, *Clostridium*, *Ruminococcus*, *Lactobacillus*	Deconjugation of glycine or taurine from primary BAs.	Unconjugated primary BAs (CA, CDCA)	[4,77,78]
*bai* operon	*Clostridium*, *Ruminococcus*	7α-dehydroxylation of primary BAs.	Secondary BAs (DCA, LCA)	[3,78,79,80]
Hydroxysteroid Dehydrogenase (HSDH)	*Bacteroides thetaiotaomicron*, *Ruminococcus gnavus*	Oxidation and epimerization of hydroxyl groups on BAs.	Diverse secondary BAs, including iso-bile acids	[80,81,82]

BAs, bile acids; CA, cholic acid; CDCA, chenodeoxycholic acid; DCA, deoxycholic acid; LCA, lithocholic acid; REF, references.

**Table 2:** Enterotype-Specific Bile Acid Metabolism.

Feature	ET-B (Bacteroides-Dominant)	ET-P (Prevotella-Dominant)	ET-R (Ruminococcus-Dominant)
Dominant Taxa [2]	*Bacteroides* spp.	*Prevotella* spp.	*Ruminococcus* spp.
BSH Activity	Highest Activity (Extensive gene repertoire) [19]	Moderate/Low Activity [25]	Intermediate Activity [19]
Key Enzymatic Step	7α-Dehydroxylation (Robust bai operon) [25]	Carbohydrate Metabolism (SCFA Production) [25]	Unique BA Modification/Sequestration [33]
Main Metabolite Pool in the Colon	High Secondary BAs (DCA, LCA) [20]	High Primary BAs (P-BAs dominate) [26]	Balanced/Unique BAs (e.g., iso-BAs, BA-polysaccharide complexes) [25]
Host Receptor Signal	Strongest FXR Activation [78,85]	Weakest FXR Activation [27,28]	Variable FXR Activation [25,36]
Overall Impact	Maximal BA Transformation and Host BA Conservation	Minimal BA Transformation and Fiber Specialization	Intermediate BA Transformation and Metabolic Stability

[ ]: references.

**Table 4:** Enterotype-Specific Metabolic Profiles and Associated Health Risks.

Enterotype/Subtype	Dominant Bacteria	Key Microbial Enzymes	Characteristic BA Profile	Health Outcome	Study Type; REF
Bacteroides 2 (ET-B2)	*Bacteroides* spp. (e.g., *B. thetaiotaomicron*)	*BSH*, Hydroxysteroid Dehydrogenases (*HSDH*s), 7α-dehydroxylases (*bai* operon)	High deconjugated BAs; elevated secondary BAs (DCA, LCA)	Obesity, Hypertension, MASLD, Colorectal Cancer, Alzheimer’s Disease, Cognitive Decline, Depression	Human (RCT); [81];
Prevotella 2 (ET-P2)	*Prevotella* spp., unclassified *Prevotellaceae* genus	Obesity, Hypertension, Metabolic Disease, Colorectal Cancer Risk	Elevated Lithocholic Acid (LCA); reduced secondary BA diversity	Obesity, Type 2 Diabetes, Intestinal Inflammation, MASH, Cirrhosis	Human (cohort); [16]
Ruminococcus (ET-R) or Lachinospira (ET-L)	*Ruminococcus* spp. (*R. gnavus*, *R. torques*), *Lachnospira*	Mucin-degrading GH; 3α-HSDH, 3β-HSDH (produces 3-dehydro-CA, Iso-CA)	Modified BAs (3-dehydrocolate, isocholate); altered BA-mucin interactions	Constipation, Mucin Barrier Dysfunction, Dyslipidemia	Human (cohort); [14,114]
IBD-associated profile	Diminished diversity of BA-modifying bacteria; often increased Enterobacteriaceae	Reduced *BSH* and *bai* operon activity; loss of BA-transforming capacity	Elevated primary BAs (CA, CDCA); reduced secondary BAs (DCA, LCA)	Inflammatory Bowel Disease (Crohn’s, Ulcerative Colitis), Intestinal Barrier Dysfunction	Review; [115];
Enterobacteriaceae type	Family Enterobacteriaceae (e.g., *Escherichia*, *Salmonella*, *Klebsiella*)	Minimal BA-modifying enzymes; nitrate reductases correlated with an inflamed gut environment	Accumulation of primary conjugated BAs; reduced secondary BA transformation	Dysbiosis, Intestinal Inflammation, Associated with IBS and IBD exacerbations	Review; [116]
Bifidobacterium type	*Bifidobacterium* spp. (*B. pseudocatenulatum*, *B. longum*)	*BSH*	Deconjugated BAs with enhanced hydrogel-forming properties	Generally protective; beneficial for gastrointestinal health and barrier function	In vitro; [80]

BAs, bile acids; CA, cholic acid; CDCA, chenodeoxycholic acid; DCA, deoxycholic acid; LCA, lithocholic acid; IBD, irritable bowel disease; IBS, irritable bowel syndrome; *BSH*, Bile Salt Hydrolase; *HSDHs*, hydroxysteroid dehydrogenases; GH, glycosyl hydrolase; MASLD, metabolic-dysfunction-associated steatotic liver disease; MASH, metabolic-dysfunction-associated steatohepatitis.

**Table 5:** Intervention Strategies, Mechanisms, and BA Targets by Enterotype.

Enterotype	Recommended Intervention	Hypothesized Mechanism of Action	Targeted BA Changes
*Prevotella*-Dominant (ET-P)	Increase protein from fish and lean meats. Include moderate healthy fats (omega-3). Reduce refined carbohydrates (white rice, white bread, sweets). Replace with low-glycemic complex carbs. Include vegetables.	Corrects protein/fish dietary deficiency observed in dysbiotic *Prevotella* states. Reduces excessive carbohydrate fermentation that promotes inflammatory *P. copri* expansion. Shifts microbiome toward healthier composition while reducing *Prevotella* dominance.	Reduced LCA production; decreased inflammatory BA metabolites; improved BA signaling through balanced microbial composition; normalized primary: secondary BA ratio.
*Bacteroides*-Dominant (ET-B)	Moderate-to-low-fat diet emphasizing unsaturated over saturated fats. Adequate lean protein (20–25% calories). Include vegetables with every meal (especially cruciferous and leafy greens). Add BA-binding soluble fibers (psyllium, oat beta-glucan, pectin). Targeted *BSH*-modulating probiotics.	Reduces fat intake to about 30 energy % and uses more MCT, which decreases bile secretion and secondary BA production (DCA, LCA). Vegetables provide polyphenols and antioxidants that mitigate BA toxicity. BA-binding fibers sequester hydrophobic BAs for fecal excretion.	Significantly reduced DCA and LCA production; decreased hydrophobic secondary BA load; improved conjugated: unconjugated ratio; reduced cancer risk and BA-mediated inflammation.
Ruminococcus-Dominant (RT-R)	Balanced macronutrient diet with adequate fiber (but not excessive). Regular consumption of fermented foods (kimchi, sauerkraut, kefir, yogurt). Include mucus-protective nutrients (omega-3, zinc, vitamin A). Increase soluble fiber and water intake to improve transit time. Prebiotics supporting beneficial commensals (inulin, FOS).	Fermented foods and probiotics improve intestinal transit time and reduce constipation associated with mucin degradation. Balanced fiber supports beneficial bacteria without overfeeding mucin degraders. Mucus-protective nutrients maintain intestinal barrier integrity despite *Ruminococcus*’s mucin-degrading activity.	Balanced primary: secondary BA ratio; prevention of BA dysregulation from mucin barrier compromise; improved BA-mediated gut motility signaling; reduced constipation-related BA retention.
Dysbiotic States (BAM)	Fecal Microbiota Transplantation (FMT) from donors with verified BA-metabolizing capacity. Post-FMT: personalized diet based on established enterotype. Low-FODMAP during acute inflammation.	Restores comprehensive BA-metabolizing functions (*BSH*, *bai* operon). Re-establishes microbial diversity and functional redundancy for secondary BA production.	Decreased serum C4 (primary BA synthesis marker); normalized secondary BA production; reduced BA malabsorption; restored conjugated/unconjugated balance.

Enterotype-based recommendations account for functional metabolic characteristics rather than bacterial composition alone. *Prevotella* in metabolic disease reflects carbohydrate excess with protein/fish deficiency requiring dietary correction. Bacteroides’ high secondary bile acid (BA) production (DCA, LCA) necessitates fat reduction and vegetable inclusion for cancer risk mitigation. ET-R is associated with mucin degradation and constipation; fermented foods, adequate hydration, and mucus-protective nutrients address these specific challenges. Individual optimization requires assessment of enterotype subtypes, host genetics, and clinical presentation.

## Data Availability

All data involved in the review paper were included in the manuscript.

## Abbreviations

ASBT, apical sodium dependent bile acid transporter; AMPK, adenosine monophosphate-activated protein kinase; BA, bile acid; BAAT, bile acid CoA:amino acid N-acyltransferase; BACS, bile acid CoA synthase; Bai, bile acid-inducible; BSH, bile salt hydrolase; CA, cholic acid; CAZyme, carbohydrate-active enzymes; CDCA, chenodeoxycholic acid; CREB, cyclic AMP response element-binding protein; CRC, colorectal cancer; CYP7A1, cytochrome P450 family 7 subfamily A member 1; DCA, deoxycholic acid; DIO2, Type 2 iodothyronine deiodinase; EHC, enterohepatic circulation; EGFR, epidermal growth factor receptor; ET-B, *Bacteroides* dominant enterotype, ET-B2—*Bacteroides* type 2 dominant enterotype; ET-L, Lachnospira-associated enterotype; ET-P, *Prevotella* dominant enterotype; ET-R, *Ruminococcus* dominant enterotype; FGF19, Fibroblast growth factor 19; FMT, Fecal microbiota transplantation; FXR, farnesoid X receptor; GLP-1, glucagon-like peptide; GPBAR1, G-protein-coupled bile acid receptor 1; HNF4α, hepatocyte nuclear factor 4α; IBD, irritable bowel disease; IBABP/FABP6, ileal bile acid-binding protein or fatty acid-binding protein 6; IL, interleukin; LCA, lithocholic acid; LXR, liver X receptor; MASH, metabolic dysfunction-associated steatohepatitis; MASLD, metabolic dysfunction-associated steatotic liver disease; NF-κB, nuclear factor-kappa B; OST-α/β/SLC51A/B, organic solute transporter; QTL, quantitative trait locus; SHP, small heterodimer partner; SLC10A2, solute carrier family 10 member 2; T2D, type 2 diabetes; TGR5, Takeda G protein-coupled receptor 5; TLR4, toll like receptor 4; TNF, tumor necrosis factor.
